# 3D Morphometric and Posture Study of Felid Scapulae Using Statistical Shape Modelling

**DOI:** 10.1371/journal.pone.0034619

**Published:** 2012-04-11

**Authors:** Kai Yu Zhang, Alexis Wiktorowicz-Conroy, John R. Hutchinson, Michael Doube, Michal Klosowski, Sandra J. Shefelbine, Anthony M. J. Bull

**Affiliations:** 1 Department of Bioengineering, Imperial College London, South Kensington, London, United Kingdom; 2 Structure and Motion Laboratory, The Royal Veterinary College, Hatfield, Hertfordshire, United Kingdom; 3 Max Planck Institute for Molecular Cell Biology and Genetics, Dresden, Germany; University College London, United Kingdom

## Abstract

We present a three dimensional (3D) morphometric modelling study of the scapulae of Felidae, with a focus on the correlations between forelimb postures and extracted scapular shape variations. Our shape modelling results indicate that the scapular infraspinous fossa becomes larger and relatively broader along the craniocaudal axis in larger felids. We infer that this enlargement of the scapular fossa may be a size-related specialization for postural support of the shoulder joint.

## Introduction

The forelimbs of Felidae (cats) play important roles in locomotion [1]–[3] and are an essential part of the prey-killing apparatus [4]. As a morphologically complex segment of the forelimb, the scapula transmits locomotor loads to the thorax, stabilises the shoulder and allows forelimb mobility; *e.g.* for climbing and prey apprehension [5]. Scapular morphology results from the complex influence of historical (phylogenetic ancestry) and functional (*i.e.*, selective) factors [3]. Furthermore, scaling and allometry studies indicate that body size can also influence scapular morphology [6], [7]. In these regards, characterising the morphology of the scapula may provide insights into locomotion patterns and the particular functional capabilities associated with phylogenetic lineages such as Felidae.

Scaling studies of limb skeletal morphology have historically focused on the long bones (especially humerus, radius/ulna, femur, and tibia) using standard measures such as diameter, length and cross-sectional parameters. Very few studies have examined scaling of the pelvis or shoulder girdle because of the difficulty of parameterizing their complex shapes [7]. With any complex shape, one must assess how particular shape parameters may be related to locomotor parameters. This assessment is fairly straightforward for long bones but is more ambiguous for the scapula. Many morphometric studies of the scapula have involved analysis based on digitising landmarks in two dimensions (2D) (*e.g.* [3], [8]–[14]). Scapulae, however, are not planar; thus some information may be lost or distorted in a 2D representation of such a 3D object [8]. Some recently reported 3D morphometric studies relied on manually identifying anatomical features as landmarks (*e.g.* [15]–[17]); the positions and numbers of landmarks varied between different studies. This method may omit critical features by only focusing on a few manually selected anatomical landmarks. Hence an approach is needed that considers the maximal amount of 3D morphological detail.

Statistical shape modelling is a model-based image analysis technique that provides a parametric framework for representing variability in a large number of individual complex anatomical shapes [18]. The basic approach used in making a statistical shape model (SSM) is to establish the pattern of ‘legal’ variation in the shape and spatial relationships of the structures in a given class of images [19]. This technique allows a 3D morphometric analysis to reveal the important shape parameters. In addition it highlights how multiple parameters change together, rather than focusing on one parameter at a time. In this study statistical shape modelling is used to determine the principle morphological variations (MVs) in the scapulae of felids.

Limb posture is an important attribute of animal locomotion because it influences the patterns of movements and muscle activity that can contribute to support, braking and propulsion [2], [20], [21]. Fischer *et al.* [2] examined limb postures of eight different mammalian species at footfall (FF), lift off and throughout a stance phase (‘amplitude’) using cineradiography. They concluded that the kinematic parameters (segment and joint angles during each stance phase) of forelimbs are independent of speed and gait, while the hindlimb kinematics varied with gaits. Day and Jayne [21] examined the limb postures at FF and midstance (MS) of nine species within the Felidae and found that the larger species of felids did not have more upright limbs than smaller species. This lack of size-related postural change in felids is dissimilar to observed postural changes in many other mammals [20] and suggests that larger felids might have evolved unusual specializations that enable a relative increase in muscular forces to maintain posture. Considering that body size, locomotor habits, and phylogenetic factors all influence scapular morphology, here we investigate how felid limb posture is correlated with 3D scapular morphology, in order to gain a greater understanding of the relationship between animal size, limb function, and scapular anatomy.

## Results

### With size model

**Figure 1:**
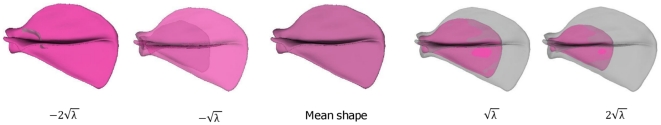
The first MVs of the ‘with size’ SSM (The mean model is in pink).

In the ‘with size’ model, the contribution by the first MV alone claims more than 99% of total variation. By visualising the model with score of 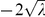 to 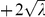 (Figure 1), it can be seen that this MV predominantly responds to scapular size. Furthermore, the change rates of parameter values (Table 1) indicate that when body size increases, the scapula also becomes broader craniocaudally, and the spine inclines caudomedially toward the infraspinous fossa.

**Table 1:** Change rates of parameter values for the first MV in the ‘with size’ SSM.

	Δ_B/L_	Δ_W/B_	Δ_W/L_	Δ_SpA_	Interpretation
**1^st^ MV (** **Figure 2** **)**	10.5%	2.0%	12.6%	7.7%	Substantial change in size of fossa relative to length, and also the inclination of spine against fossa.

### Without size model

**Figure 2:**
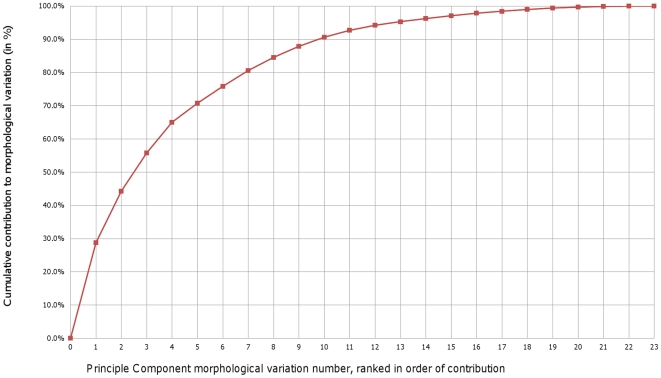
The cumulative percentage of contributions by each MV in the ‘without size’ SSM.

The cumulative percentage of contributions by each MV in ‘without size’ SSM is shown in Figure 2.

**Figure 3:**
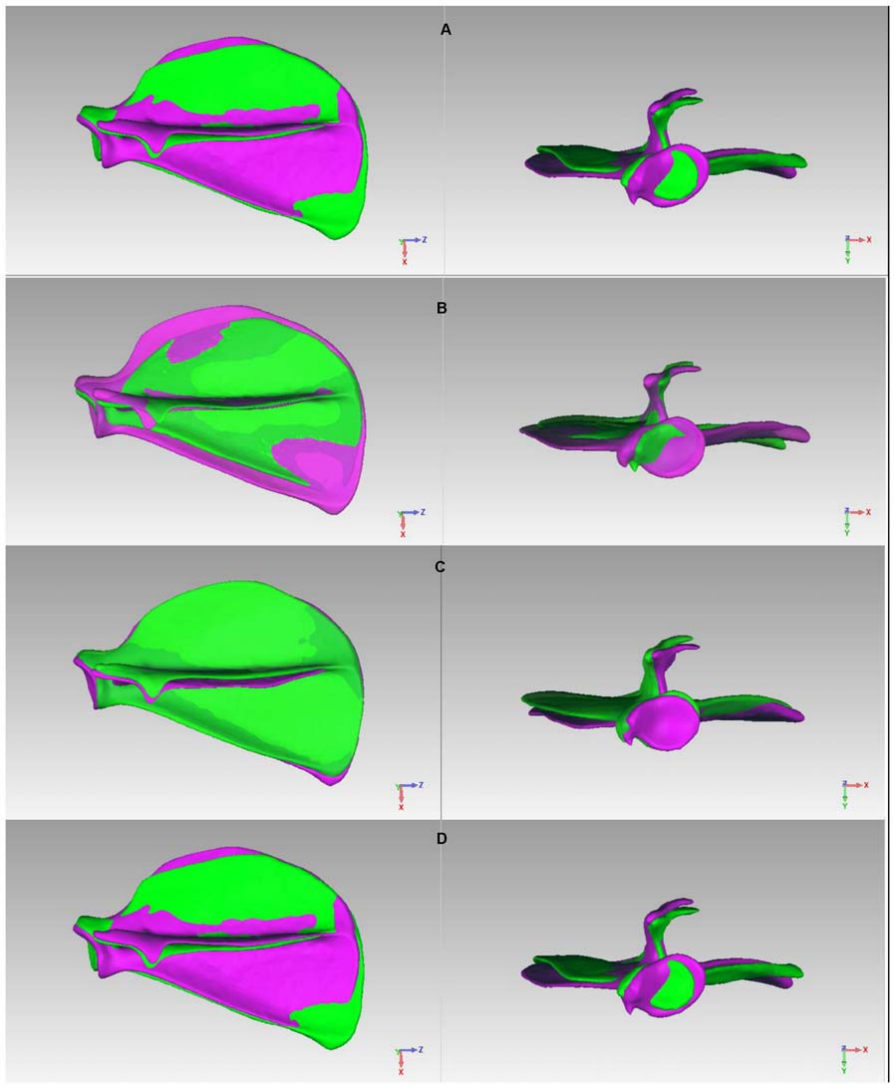
The first four MVs of the ‘without size’ SSM (model with 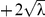
 **is in purple; model with** 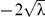 **is in green).** A: 1^st^ MV; B: 2^nd^ MV; C: 3^rd^ MV; D: 4^th^ MV.

In the ‘without size’ model, the first six MVs contribute more than 75% of the total variation. The fifth and sixth MVs are complex and difficult to describe qualitatively. The first four MVs of the ‘without size’ SSM are shown in Figure 3.

The change rates of parameter values for each MV in the ‘without size’ SSM are shown in Table 2.

**Table 2:** Change rates of parameter values for the first four MVs in the ‘without size’ SSM.

	Δ_B/L_	Δ_W/B_	Δ_W/L_	Δ_SpA_	Interpretation
**1^st^ MV (** **Figure 3a** **)**	13.6%	13.5%	27.2%	−0.9%	Size change of fossa, particularly the infraspinous fossa.
**2^nd^ MV (** **Figure 3b** **)**	22.2%	2.1%	24.4%	−1.5%	Size change of fossa, both supraspinous and infraspinous fossae.
**3^rd^ MV (** **Figure 3c** **)**	9.3%	0.4%	9.4%	14.1%	Inclination of spine against fossa.
**4^th^ MV (** **Figure 3d** **)**	6.3%	−6.0%	0.2%	−5.8%	Size change of fossa, particularly the supraspinous fossa.

### Posture and moment arm (MA) correlation

No significant correlation was found between the humerus angles relative to vertical (θ) and the score of each species on each MV in either posture datasets.

The results of the MA correlation test (see Methods) are consistent in both posture datasets, and are shown in Table 3 (only those results with statistical significance are included).

**Table 3:** Results of correlation tests between MAs and MVs in the ‘with size’ and ‘without size’ SSMs.

	T_1_ at FF (MAdataset1*)		Interpretation
	r^2^	p	
**1^st^ MV in ‘with size’ SSM**	0.833	0.002	Larger species of felids have a larger MA of the vertical component of the GRF at FF.
**1^st^ MV in ‘without size’ SSM**	0.556	0.037	Those species with a relatively broader infraspinous fossa have a larger MA of the vertical component of the GRF at FF.

	T_2_ at FF (MAdataset1)		Interpretation
	r^2^	p	
**1^st^ MV in ‘with size’ SSM**	0.889	0.001	Larger species of felids have a larger MA of the horizontal component of the GRF at FF.

	T_2_ at MS (MAdataset1)		Interpretation
	r^2^	p	
**1^st^ MV in ‘with size’ SSM**	0.889	0.001	Larger species of felids have a larger MA of the horizontal component of the GRF at MS.

	T_2_ at MS (MAdataset2**)		Interpretation
	r^2^	p	
**1^st^ MV in ‘with size’ SSM**	0.867	0.015	Larger species of felids have a larger MA of the horizontal component of the GRF at MS.

* Posture data reported by Day and Jayne (2007);

** posture data collected by Wiktorowicz-Conroy *et al.* (manuscript in review).

## Discussion

Two types of landmarks have been commonly used in previous scapular morphometric studies: biological landmarks (type1) which describe discrete positions of tissues or structures (often as single points), and morphometric landmarks (type2) which describe curvature or outlines. Due to the complexity of scapular structure, type1 landmarks are nearly absent [3] and often both types of landmarks have been adopted, although type2 landmarks may not be located in the same anatomical location [22]. A conventional method in scapular morphometric studies has been to take dimensional measurements, such as distance or angles between the landmarks (*e.g.* [1], [9], [23]), although by fixing the variables in advance, the hypothesis to be tested has been presumed and therefore important information could be missed. For example, Zelditch *et al.* [22] argued that this method could not be used for phylogenetic analyses because ‘manipulation of variables chosen in advance of analysis limits the possibility of assessing detailed similarity’ due to the fact that these variables were expected to differentiate species. However, identifying similarity is as important as, if not more important than difference for phylogenetic analyses. Landmark-based studies of scapulae have also often involved canonical variate analysis (CVA) and/or principal component analysis (PCA) (*e.g.* [14]–[17]). The number and position of landmarks are critical in CVA and PCA analysis. By selecting landmarks in advance, they could fail to align along the principal axis and therefore important variations could be missed. To avoid these disadvantages, geometric methods such as a thin-plate spline decomposed by its partial (*e.g.* [8], [12], [22]) or relative warps analysis [3] have been adopted in some scapular morphometric studies. Most of these studies were based on 2D data, which thus could not capture the 3D complexity of scapular shape. The number and positions of landmarks also varied, making it difficult to determine whether landmark selection affects analysis. Therefore, the method adopted in this study allows automatic selection of surface points covering the whole scapula, avoiding human manipulation in describing the shape. In this way, the maximum amount of 3D shape information can be preserved to allow more accurate 3D analysis. This comparative method potentially could also be used in phylogenetic analysis to reveal both similarities and differences.

**Figure 4:**
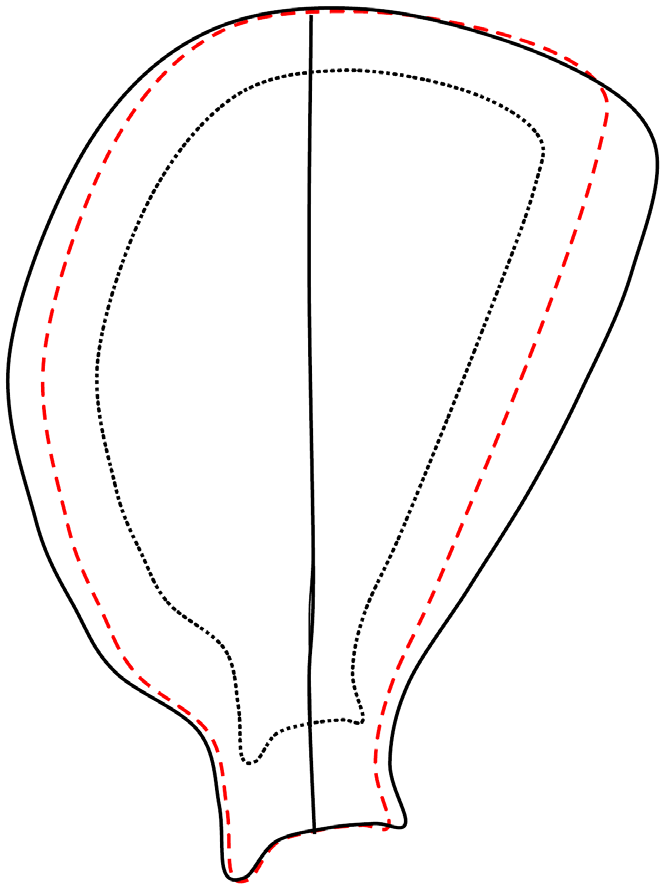
Sketches of scapular fossa when changing size uniformly and accordingly as the first MV of the ‘with size’ SSM. The original fossa is in dashed black; the fossa by changing size uniformly is dashed red, and the fossa by changing size accordingly as the first MV of the ‘with size’ SSM is in solid black.

**Figure 5:**
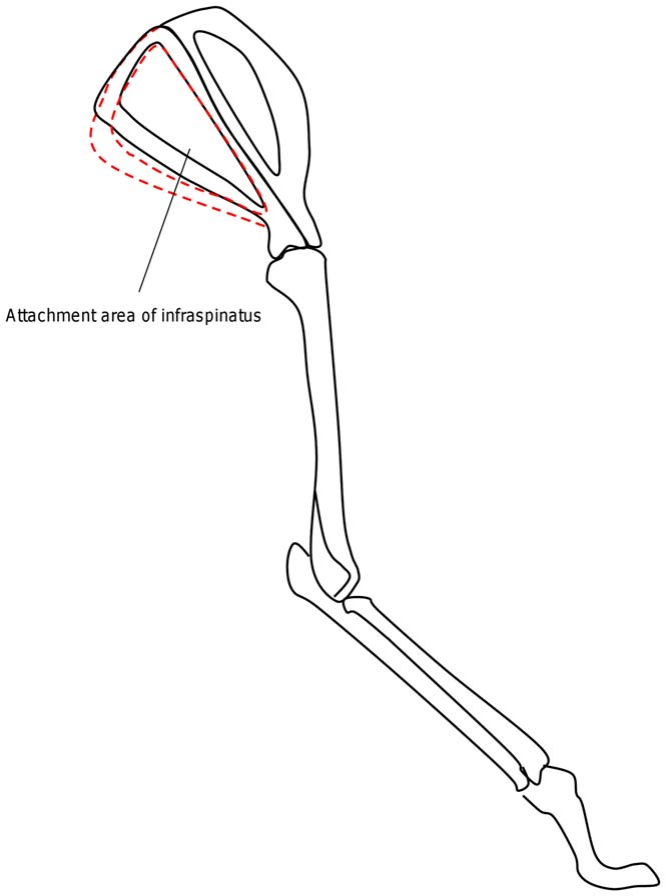
Sketch of a forelimb with different sizes of infraspinous fossa (dotted red sketch is for larger animal).

Furthermore, the statistical shape modelling method highlights how multiple 3D shape parameters change together, which is more informative than measuring single variations. For example the first MV of the ‘with size’ SSM indicates that as body size increases, the infraspinous and supraspinous fossae become craniocaudally broader (see Figure 4). This can be understood in the light of the study by Day and Jayne [19] showing that felid postural scaling is dissimilar from most other mammals' in that limb posture is maintained with increasing body size. Here we have shown that the scapular morphology is not preserved with size. The broadening of the fossae suggests increases in sizes of the attaching muscles, such as infraspinatus (see Figure 5) which support the maintenance of limb posture in gait by resisting a moment that would protract the humerus. The size or shape variations of other muscles attached close to the infraspinous fossa, such as teres major, subscapularis and rhomboideus, are difficult to assess in this study as they are attached to the caudal and dorsal borders of the scapula where our SSM did not show significant variations. Resolving this matter will require further acquisition and analysis of muscle data through dissection or imaging.

The complex shape of the scapula makes it difficult to use traditional scaling approaches. A common approach in scaling studies is to use linear measurements of bone dimensions, such as length and midshaft diameter of long bones [24]–[41], because they are easily obtained and are thought to have strong relationships with factors such as body mass [24]–[27], locomotor pattern [25], [27]–[29], body shape [25], [26], [30], [31] or posture [32], [33]. Cross-sectional area and bone curvature are also assessed to quantify strength-mass relationships [26], [34]–[39]. Previous scaling and morphology studies involving felids have demonstrated that the lengths of long bones (tibia and ulna, humerus and femur) scale isometrically with body mass, particularly for the tibia and ulna [40], [41]. Humeral circumference on the other hand, appears to increase allometrically with body mass [4], [39], [41]. Unfortunately, none of these approaches or measurements can be easily transferred to the scapula, yet scapula morphology is important because it is distinct from long bones in its functions and anatomical connections. The multiple variations accompanied by size change revealed in the first MV of the ‘with size’ SSM also indicates that using a single scaling factor to scale the scapula uniformly is not sufficient to capture the complex variability related to size change in scapulae. Instead, within the species we used to construct the SSM, it would be more accurate to scale the scapulae by varying the score of this MV. This shows that the 3D statistical shape modelling method potentially can be used to provide a more accurate scaling solution than to use a single scaling factor.

No correlation has been found between the angle of the shoulder joint and the first MV of the ‘with size’ SSM, which predominantly responds to scapular size. This bolsters the conclusion drawn by Day and Jayne [21] that larger species of felids do not have more extended limbs than smaller species.

Previous studies have suggested that the ground reaction force (GRF) in quadrupeds tends to be aligned with the limb axis during steady speed locomotion [42], [43]. In this study we did not measure the actual GRF moment arms (MAs) using the limb axis as an approximation of the GRF vector (or better yet, using actual experimental data on GRF), but rather we estimated the resolved horizontal and vertical components of the GRF. We did this because the species in this study cover a broad body size range. Hence these resolved GRF components would provide more information by isolating the MA of the vertical component; which is not dependent on the shoulder height; from the horizontal component; which is dependent on the shoulder height. Not surprisingly, the first MV of the ‘with size’ SSM is correlated with the horizontal MA. The MA of the vertical component of the GRF at FF is also correlated with size, which is expected because the posture is conserved across felid species. Interestingly, the MA of the vertical component of the GRF at FF is also correlated with the size of the infraspinous fossa (first MV of the ‘without size’ SSM), suggesting that a larger muscle mass is required to maintain shoulder joint postures in larger species.

### Conclusions

3D statistical shape modelling was used to extract morphological variation parameters from 43 specimens of 23 felid species. The predominant variation of scapular shape across the species examined in this study is size-related. However, the scapulae also become craniocaudally broader as body size increases. The moment arm of the vertical component of the GRF at footfall is correlated with both body size and the size of the infraspinous fossa, which indicates that larger species of felids intend to have a larger infraspinous fossa to better support their own weight about the shoulder joint.

## Materials and Methods

### Statistical Shape Modelling

A set of 3D SSM was built from a training set of 3D surfaces that were aligned to a common coordinate frame. Each surface model in the dataset was represented by landmarks and correspondences were established between these landmarks to ensure all surfaces were represented in the same way. The term ‘landmark’ can be any feature that describes the 3D surface, such as a line feature or an area feature [44]. In this study, arbitrary points on the surface were adopted as landmarks.

**Figure 6:**
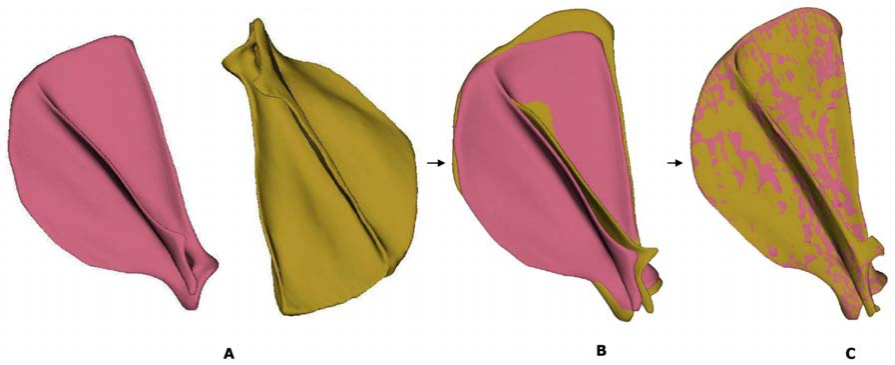
Establishing correspondences between reference (in pink) and target shape (in yellow). A: original position; B: align reference and target shapes; C: reference shape deformed to target shape.

A scapula in the dataset was randomly chosen as the reference shape. The iterative closest point (ICP) algorithm proposed by Besl and McKay [45] was employed to align all target surface models to the coordinate system defined by the reference shape (Figure 6b). Then a non-rigid surface-based registration using the multiresolution free-form deformation (FFD) algorithm proposed by Rueckert *et al.* [46] and further extended by Schnabel *et al.* [47] was used to establish correspondences within the dataset. This non-rigid surface-based registration was proposed based on B-splines, which are a type of mathematical model commonly used in computer graphics for generating and representing curves and surfaces. By changing the number and position of control points, B-splines can be locally deformed, and therefore curves or surfaces constructed from a number of B-splines can be deformed. This allows a target shape surface to be embedded onto a volumetric mesh, which initially derives from the reference shape and defines a continuous deformable field: *i.e.* the initial control points of this mesh were surface points of the reference shape. The mesh is subsequently subdivided into higher resolution levels by inserting control points into the current level of control points and decreasing the mesh space [48]. The multiresolution FFD algorithm generates a hierarchy of deforming to deform the mesh by translating a sequence of control points, and minimizes the distance between every surface point on the reference shape and its closest point on the target shape. The optimal control point values are then calculated using the algorithm proposed by Lee *et al.* [49]. This process is repeated until the distances between the mesh and the target shape cannot be minimized any further. In this study, after the registration, the deformable mesh was deformed to the target shape. For each surface point on the reference shape, its closest point on the target shape was then assumed to be its corresponding point (Figure 6c).

After establishing correspondences among the dataset, PCA was performed on the matrix formed by all surface models to extract the principal axes to describe the morphological variability.

### Experimental Dataset

CT or X-ray microtomography (XMT) images of 43 scapulae from 23 felid species were used to construct the SSM. The sample information is listed as supporting information (Table S1). Specimens were selected to provide a wide range of body masses and provide broad coverage of felid taxa and their locomotor specialisations. Larger scapulae were imaged in clinical CT scanners (LightSpeed16, GE MEDICAL SYSTEMS Ltd, UK; Mx8000 IDT 16, Philips, Netherlands; PQ5000, Marconi Medical Systems, Inc, USA.) and smaller scapulae imaged in XMT (X-Tek HMX ST 225, Nikon Metrology Ltd, Tring, UK), due to the extremely thin flat parts of the bone in small cats. The CT images were manually segmented and finalised using Mimics (Materialise NV, Belgium) and Geomagics Studio (Geomagic, Inc., USA) to achieve good quality 3D surface models.

**Figure 7:**
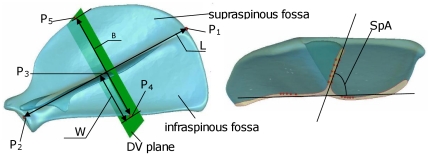
Reference features and parameters used to quantify MVs. P_1_: intersection point of spine and dorsal border; P_2_: centre of glenoid cavity; P_3_: mid-point between P_1_ and P_2_; P_4_: intersection point of caudal border with DV plane; P_5_: intersection point of cranial border with DV plane.

The dataset in this study includes species across a broad body mass range; roughly 1–300 kg. Therefore two types of models were constructed to examine the variability: the ‘with size’ SSM was constructed from scapulae in their original size; and the ‘without size’ SSM was constructed from scapulae scaled to a reference shape to minimize the size variation in the dataset. The scaling factor (SF) in the ‘without size’ SSM for each shape model was calculated as: 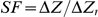; in which 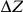 is the distance between P_1_ (the intersection point of the scapular spine and the dorsal border) and P_2_ (the centre of the glenoid cavity) of the scapula (Figure 7) to be scaled, and 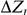 is the distance between P_1_ and P_2_ of the reference scapula.

### Data Analysis

The MVs extracted from the datasets were exported both qualitatively and quantitatively. The following reference features and parameters were constructed and measured for each variation (Figure 7):

- Length (L): the distance between the intersection point of the scapular spine and the dorsal border (P_1_) and the centre of the glenoid cavity (P_2_);
- Dorsal-ventral (DV) plane: the plane through the mid-point (P_3_) between P_1_ and P_2_, and perpendicular with the line via P_1_ and P_2_;
- Breadth (B): the distance between the intersection point of the caudal border with the DV plane (P_4_) and the intersection point of the cranial border with the DV plane (P_5_);
- Infra-breadth (W): the distance between P_3_ and P_4_.
- SpA: angle between lines fitted through landmarks selected from the spine and fossa borders after truncated by the DV plane.

For each MV, the score on that principal axis was varied from 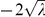 to 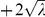 (where 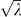 is the standard deviation along each principal axis) with the scores on all other principal axes fixed as 0 to observe the variability. In particular, the values of parameters were measured on the models with score of 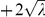, 0 and 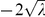 along each principal axis. B/L, W/B and W/L were calculated for each MV to characterise the size changes of the infraspinous and supraspinous fossae relative to the length. The change rates of these parameters were calculated as: 
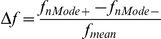
In which 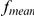 is the parameter value of the model with score of 0; 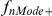 is the parameter value of the model with score of 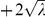, 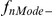 is the parameter value of the model with score of 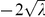.

### Correlation with posture and MA data

**Figure 8:**
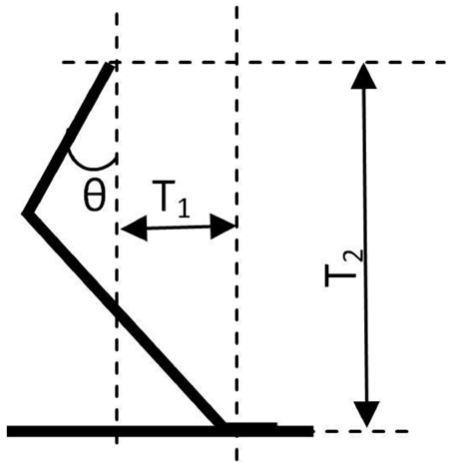
Moment arm from the most proximal point of humerus to the presumed vertical vector of the ground reaction force (GRF measured at FF).

Day and Jayne [21] reported kinematic data on limb postures for walking felids. The nine species they examined included domestic cat, serval, ocelot, lynx, leopard, cheetah, cougar, lion and tiger. We reconstructed the postures of these species using the mean values of joint angles of forelimbs at footfall (FF) and mid-stance (MS), and the mean relative length of each segment (% total limb length) that were reported by Day and Jayne [21]. The total limb length was scaled using the same scaling factor for constructing the ‘without size’ SSM for each species. The moment arms (MAs) of the presumed vertical component (T_1_) and of the horizontal component (T_2_) of the GRF from the most proximal point of the humerus at FF and MS were estimated instead of the true GRF data. The vertical component corresponds to the force acting in line with gravity, and the horizontal component corresponds to the propulsion or braking force component. The correlations between the MA at FF and MS and the MV of each species on each principal axis were tested using Spearman test at a 95% confidence level. The correlations between humerus angles relative to vertical (θ) (Figure 8) at each stance and the score of each species on each principal axis were also tested.

In addition, we also tested the correlations between the score of each species on each MV and the posture data taken by Wiktorowicz-Conroy *et al.* (manuscript in review) from six species of felids (domestic cat (*Felid catus*; N = 2), ocelot (*Leopardus pardalis*; N = 1), caracal (*Caracal caracal*; N = 2), leopard (*Panthera pardus*; N = 2), serval (*Leptailurus serval*; N = 2), and tiger (*Panthera tigris*; N = 4)). Kinematics of the forelimb joints (metacarpal-phalangeal, wrist, elbow, shoulder) were obtained at MS during low speeds (walks). Joint angles and segment lengths of the forelimb were determined using methods consistent with previous studies [20], [50]–[54].

## Supporting Information

Table S1 **Information of the dataset used to construct the SSM of cat scapulae is listed in this table.**
(DOC)
