# Supplementary material for: 3D Morphometric and Posture Study of Felid Scapulae Using Statistical Shape Modelling
Source: PLoS One. 2012 Apr 11;7(4):e34619. doi: 10.1371/journal.pone.0034619 (PMC3324489; doi:10.1371/journal.pone.0034619)
Supplement: Table S1 — Information of the dataset used to construct the SSM of cat scapulae is listed in this table. (DOC) [file pone.0034619.s001.doc]

**3D morphometric and posture study of felid scapulae using statistical shape modelling**

**Supporting Information Table S1**

| **Species** | **No. of scapulae** | **Binomial name** | **body mass (kg)** | **Museum** | **accession** |
| --- | --- | --- | --- | --- | --- |
| **Serval** | 2* | *Leptailurus serval* | 7-12 | NHM  NHM | 133e  1966.7.11.1 |
| **Jaguarundi** | 2* | *Puma yagouaroundi* | 3.5-9.1 | NHM  NHM | 1964.4.22.1  1965.2.2.1 |
| **Ocelot** | 2* | *Leopardus pardalis* | 8-18 | NHM  NHM | 1862.3.19.15_1232.d  1952.1083 |
| **Cougar** | 2* | *Puma concolor* | 29-100 | UMZC | K5741 |
| **Leopard** | 2* | *Panthera pardus* | 23-91 | NHM  NHM | 1880.2.16.1  1940.1.20.18 |
| **Tiger** | 3* | *Panthera tigris* | 75-306 | NHM  NHM  RVC | 1978.2640  1851.5.5.2  Tiger1 |
| **Leopard cat** | 2* | *Prionailurus bengalensis* | 0.55-3.8 | NHM  NHM | 1858.12.1  1860.4.23 |
| **Snow leopard** | 2* | *Uncia uncia* | 27-55 | NHM  NHM | 1962.12.11  1967.6.29 |
| **Oncilla** | 1 | *Leopardus tigrinus* | 1.5-3 | NHM | 63.1210 |
| **Caracal** | 2* | *Caracal caracal* | 13-18 | NHM  NHM | 1855.9.17.2.981e  1982.493 |
| **Lynx** | 2* | *Lynx lynx* | 18-30 | NHM  NHM | 1960.8.4.2  1985.803 |
| **Jaguar** | 1 | *Panthera onca* | 56-96 | NHM | 1858.5.26.9 |
| **Cheetah** | 2* | *Acinonyx jubatus* | 36-65 | NHM  NHM | 1940.1.20.17  1991.588 |
| **Lion** | 3* | *Panthera leo* | 120-250 | UMZC  NHM  NHM | K5466  1935.9.19.5b  1952.11.13 |
| **Clouded leopard** | 2* | *Neofelis nebulosa* | 15-23 | NHM  NHM | 1854.6.14.2  1952.10.20.8 |
| **Domestic cat** | 4* | *Felis catus* | 3-5 | NHM  RVC  RVC  RVC | 1952.10.20.4  Felis_catus_1  Felis_catus_2  Fctus21 |
| **Geoffroys cat** | 1 | *Leopardus geoffroyi* | 2-5 | NHM | 1932.2.14.1 |
| **Flat headed cat** | 1 | *Prionailurus planiceps* | 1.5 – 2.5 | NHM | 1864.8.17 |
| **Margay** | 1 | *Leopardus wiedii* | 2.6-4 | NHM | 1932.2.14.1 |
| **Jungle cat** | 1 | *Felis chaus* | 3-12 | NHM | 1892.5.22.1 |
| **Fishing cat** | 2* | *Prionailurus viverrinus* | 5-16 | NHM  NHM | 1860.7.22.22  1975.2284 |
| **African golden cat** | 1 | *Profelis aurata* | 8-16 | NHM | 1965.8.26.3 |
| **Canadian lynx** | 1 | *Lynx canadensis* | 8-11 | UMZC | K6682 |

*: an average surface model was calculated using all scapulae.

RVC: Royal Veterinary College, London, UK.

UMZC: University Museum of Zoology, Cambridge, UK.

NHM: Natural History Museum, London, UK.

**Table S1 Information of the dataset used to construct the SSM of cat scapulae.**
